# An optimistic point in COVID-19 pandemic: a case report of large adult congenital cystic adenomatoid malformation

**DOI:** 10.1186/s43055-021-00439-8

**Published:** 2021-02-17

**Authors:** Mohamed rafi Kathar Hussain, N. Kulasekeran, A. M. Anand

**Affiliations:** Department of Radiodiagnosis, Sri Manukula Vinayagar Medical College, Kalitheerthalkuppam, Puducherry, India

**Keywords:** CCAM, CT, Lung, Congenital, Metaplasia

## Abstract

**Background:**

Covid-19 pandemic is a major health calamity causing global crisis involving every aspect of the society. CT chest has become an essential diagnostic investigation and as a prognostic tool for assessment for COVID-19 bronchopneumonia. This case report is about an incidental unexpected finding in a young female, who underwent CT chest screening with suspicion of COVID-19 bronchopneumonia.

**Case presentation:**

A 29-year-old female came with the complaints of sore throat, myalgia, and fever for the past 3 days. She was referred to our department for plain screening CT chest to rule out COVID 19 infection. She was an active sports person since childhood. CT chest revealed a large well-defined bullous cystic lesion of size 16 × 9.5 × 9.5 cm in the left lung lower lobe with partial sparing of its superior, anterior, and posterior basal segments. Imaging diagnosis of large bullous cystic lesion with emphysematous changes was made. No features of COVID 19 bronchopneumonia. Thoracoscopy-guided lobectomy was done, and tissue was sent to histopathological examination. Final diagnosis was large type 1 congenital cystic adenomatoid malformation with mucinous metaplasia. Our case is unique in the sense that large adult CCAM with mucinous metaplasia of the epithelium is a rare presentation. Further it was diagnosed as a part of COVID 19 screening.

**Conclusion:**

CCAM presentation in adult is rare. Asymptomatic CCAM lesion of this size diagnosed during COVID 19 chest CT screening was rarely described.

## Background

Covid-19 pandemic is a major health calamity causing global crisis involving every aspect of the society. CT chest has become an essential diagnostic investigation and as a prognostic tool for assessment for COVID 19 bronchopneumonia [1]. Practically, it also acts as screening tool in certain clinical conditions, for diagnosing COVID, when PCR is indeterminate or patient having persistent symptoms despite negative PCR [2]. Screening CT done to rule out COVID bronchopneumonia has led to the discovery of other unrelated pathologies [3]. Screening CT chest was not routinely performed previously in developing countries like India. This case report is about an incidental finding in a young female patient who underwent CT chest with suspicion of COVID 19 bronchopneumonia.

## Case presentation

A 29-year-old female came with the complaints of sore throat, myalgia, and fever for the past 3 days. She had a contact history with a known COVID patient. PCR test was negative done a day before. With persistent symptoms, she was referred to our department for the screening plain CT chest. She was an active sports person since childhood. She did not have any significant previous surgical or medical history apart from having primary tuberculosis in childhood. For this, she was treated with anti-tubercular therapy and cured. Previous chest radiographs are not available at present. Rest of the clinical history is unremarkable.

**Fig. 1:**
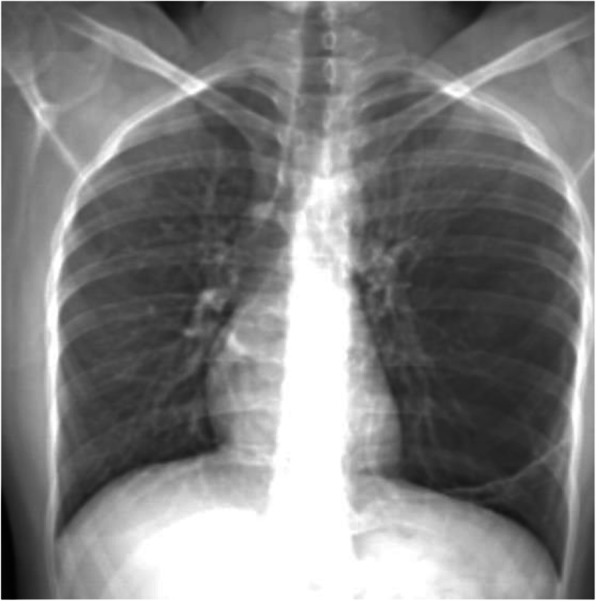
CT scout radiograph showed increased radiolucency involving mid and lower zones of the left lung with displacement of broncho-vascular markings with mild mediastinal to right side

CT scout showed increased area of radiolucency involving mid and lower zones of the left lung which is causing peripheral displacement of broncho-vascular markings. Mild mediastinal shift to the right side was seen. There is no evidence of consolidation or pneumothorax (Fig. 1).

**Fig. 2:**
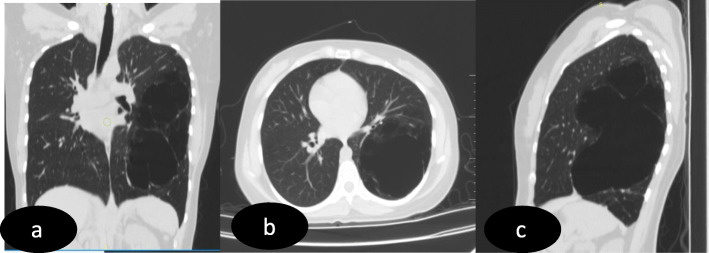
**a** Coronal, **b** axial, and **c** sagittal images of the CT chest show large bullous lesion in the left lung with few tiny internal septations and no evidence of fluid level/collection

CT chest revealed a well-defined, large bullous cystic lesion of size 16 × 9.5 × 9.5 cm in the left lung lower lobe with partial sparing of superior, anterior, and posterior basal segments. It also shows few tiny internal septations within. Anterior and medial aspect of lesion is surrounded by the normal lung parenchyma without any demarcation. Inferiorly a thin wall (of thickness ~3mm) is noted between the lesion and lung parenchyma. Laterally, the lesion is extending up to sub-pleural surface. There is no evidence of fluid level or collection within the lesion. There are associated subtle emphysematous changes seen in the postero-basal segment of left lung lower lobe. No CT features of COVID 19 bronchopneumonia. Mediastinal shift was seen in the form of displacement of trachea, mediastinum, and anterior junctional line towards right. There is no other significant abnormalities noted in the remaining part of the left lung. Right lung is normal. There is no evidence of bronchiectasis, cystic lesion, or mass in the right lung (Fig. 2 a–c).

Provisional imaging diagnosis of large bullous cystic lesion with emphysematous changes was made, since she did not have any previous respiratory complaints other than recent mild discomfort during exercise. She was advised surgical management by respiratory physician. Thoracoscopy-guided lobectomy was done outside the institute and tissue specimen was sent to histopathological examination. Macroscopic description of the specimen was resected left lung lower lobe which shows collapsed cystic space with multiple internal septation. Microscopic image shows large irregular cystic space lined by ciliated columnar epithelium with foci of mucinous metaplasia of the epithelium, and it is extending into the adjacent alveolar space in the lipidic fashion. Associated fibrous tissue was noted along the wall of the lesion. Adjacent surrounding lung parenchyma and bronchial line are normal. Final diagnosis was large type 1 congenital cystic adenomatoid malformation (CCAM) with small foci of bronchioalveolar carcinoma—mucinous type. Postoperative period was uneventful. Patient is symptom free for the past 3 months.

Our case is unique in the sense that CCAM presenting in adults is rare. And size of this CCAM is the largest, and it also shows features of malignancy. Further, it was diagnosed incidentally as a part of COVID screening. Although benefits of screening were widely known and practiced among the European countries, still screening CT chest is not routinely practiced in developing countries.

## Discussion

Congenital cystic adenomatoid malformations (CCAM) are a hamartomatous lesion of the lung due to the developmental abnormality. Etiology is unknown. It is non-hereditary disorder [4]. The estimated incidence is 1 in 25,000 to 35,000 pregnancies [4, 5]. It represents 95% of the congenital lung lesions and 25% of the congenital lung malformations [5]. It is also called as congenital pulmonary airway malformation [5, 6]. It was first identified by Stoerk et al. in 1897 and described as a congenital lung disease in 1949 by Chi’n Tang [5, 7]. It is due to arrested development of the lung during pseudo-glandular and saccular period in 7 to 35 weeks of intrauterine life [4, 5]. No sex or side predilection was seen [4, 5]. Single lobar involvement is more common than multi-lobar involvement. Lower lobe involvement is more common than the rest of the lobes [4]. Bilateral or larger lesion involvement can lead to cardiovascular compromise in fetus [5]. Most of the cases are detected by antenatal USG. At least 90% of the cases are diagnosed within 2 years [4]. Presentation in the adults is rare.

They are classified into 3 types based upon the Stocker et al. in 1977 and this was further expanded into 5 types in 2002 [5, 6, 8], which is mentioned in Table 1. Clinical presentation in case of neonates is respiratory distress. In adults, it will usually present as recurrent nonresponding chest infection [5]. Rarely as recurrent pneumothorax [7].

**Table 1:** Stocker classification of congenital cystic adenomatoid malformation [4, 5, 8]

Types	Percentage of occurrence	Morphology	Histopathology	Association
0	1–3%	Solid appearance with small and firm lungs	Tracheobronchial origin. Microscopically shows bronchiolar type airway with cartilage, smooth muscle, and glands separated by abundant mesenchymal tissue.	Differential diagnosis: bronchogenic cyst
1	50–70%	Single or multiple large cysts (> 2 cm)	Wall contains flattened, cuboidal cells. It also contains prominent smooth muscle and elastic tissue. Sometime mucus producing cells are seen and presence of cartilage is very rare	Contralateral mediastinal shift
2	15–30%	Multiple small cysts (< 2 cm)	Cyst wall lined by ciliated cuboidal to columnar epithelium that resembles respiratory bronchioles. Distended alveoli are present between the epithelium lined cyst. Mucus cells and cartilage are not seen.	Systemic anomalies like Bilateral renal agenesis, abdominal wall defects, hydrocephalus, spinal deformities, diaphragmatic hernia, jejunal atersia, tracheoesophageal fistula, imperforate anus, ventriculoseptal defects, tetralogy of Fallot, truncus arteriosis, and sirenomelia
3	5–10%	Bulky non-cystic lesions	Bronchial-like structures are lined by ciliated cuboidal epithelium and separated by masses of alveolus-sized structures by non-ciliated cuboidal epithelium .	Contralateral mediastinal shift. Rarely associated with esophageal cyst.
4	10–15%		Distal acinar origin. Large cysts (> 10 cm) lined by flattened epithelium and resting on loose mesenchymal tissue	Differential diagnosis: pulmonary blastoma

USG features are multiple varying cystic lesions in the lung with or without mediastinal shift [5]. Two measurements are taken in the antenatal USG in case of CCAM. First one is fetal lung mass size which is calculated by taking a single largest measurement of lung mass at the maximum diameter [5]. Any fetal lung mass size which is less than 5.2cm will have a good prognosis compared to those above 5.2cm. Next one is CCAM volume ratio (CVR) which is calculated by measuring the lesion in three perpendicular planes and calculating the volume and dividing by head circumference in centimeter [ CVR = (L × B × W × 0.52/HC)]. CVR more than 2 will have worst prognosis [5]. If the volume of the CCAM is more, it will lead to cardiovascular compromise and fetal heart failure.

Chest radiograph shows multiple varying size thin walled cystic spaces which are filled with air or secretions [8]. CT will provide better details when compared to chest radiograph. There are multiple well-defined varying size cystic lesions involving the whole or part of the lobe with or without associated trans-mediastinal herniation. Presence of fungal ball with associated crescent of air was also seen in few cases of superadded fungal infection [9].

The usual differential diagnosis includes intralobar sequestration, lung cyst, lung abscess, CDH, and CLE [4, 5].When compared to sequestration which derives the blood supply from the aberrant systemic circulation, CCAM has normal blood supply from the pulmonary circulation and will have patent tracheobronchial connection. Pulmonary inflammatory pseudotumor is also one of the rare differentials [6]. Co-existence of CCAM with sequestration, bronchial atresia has also been reported in the literature [7]. The common differential diagnosis in case of adult patients, when the cyst is unilocular, is intrapulmonary cyst, intralobar sequestration, and lung abscess [4].

The treatment of choice for the adult CCAM is surgical excision [4, 6]. In case of CCAM diagnosed in antenatal USG, there are different types of management based upon the case to case basis. Few cases have also been reported in the literature in which lesions disappear spontaneously on its own [5]. Early surgical management of the asymptomatic patient will have better prognosis compared to surgical management of symptomatic patient in later stage [6].

The non-neoplastic complications include recurrent pneumonia, abscess, pneumothorax, fungal colonization, and hemoptysis [4, 5, 9]. Neoplasms that can arise from the CCAM are bronchioloalveolar carcinoma, pulmonary blastoma, squamous cell carcinoma, and rhabdomyosarcoma [4]. The incidence of malignant transformation in CCAM is 1 to 3% [6]. They usually have worst prognosis [5], especially when it occurs in young adults [4]. Adult patients show frequent malignant complications like alveolar carcinoma and adenocarcinoma than pediatric patients [7].

In addition to the histopathological features mentioned in Table 1, previous literature mentions that absence of inflammatory changes in CCAM is characteristic, but recent research states that surgery done in CCAM after puberty may show signs of inflammation. Our case does not show any features of inflammation surrounding the lesion on histopathology. Thyroid transcription factor 1 (TTF1) positivity will be seen in case of lung adenocarcinoma developing from the CCAM [4]. Further it has been postulated that abnormalities in the expression of TTF-1 may lead to the development of CCAM.

On imaging, developmental anomaly of lung can be mistaken for neoplasm in an adult. Incidental detection of CCAM in asymptomatic adult patients has increased due to advent of wide availability and usage of CT. Management of adult CCAM differs when compared to that of pediatric patients [10].

## Conclusion

To conclude, the adult CCAM is rare. Larger asymptomatic lesion of this size was rarely described in literature. And this was incidentally detected in CT, which was done with suspicion of COVID bronchopneumonia in this adverse time of COVID 19 pandemic. This developmental anomaly should not be mistaken for neoplasm in adults. Further propensity of CCAM to develop in situ neoplastic lesion should also be kept in mind. Although COVID 19 is causing devastating effect all around the world, but for this young female patient, it is an optimistic event in her life.

## Data Availability

Available

## Abbreviations

COVID-19: 2019 novel coronavirus
CT: Computed tomography
CCAM: Congenital cystic adenomatoid malformations
PCR: Polymerase chain reaction
USG: Ultrasonography
CVR: CCAM volume ratio
CDH: Congenital diaphragmatic hernia
CLE: Congenital lobar emphysema
TTF: Thyroid transcription factor
